# Outcomes of laboratory-confirmed SARS-CoV-2 infection during resurgence driven by Omicron lineages BA.4 and BA.5 compared with previous waves in the Western Cape Province, South Africa^⁎^

**DOI:** 10.1016/j.ijid.2022.11.024

**Published:** 2023-02

**Authors:** Mary-Ann Davies, Erna Morden, Petro Rousseau, Juanita Arendse, Jamy-Lee Bam, Linda Boloko, Keith Cloete, Cheryl Cohen, Nicole Chetty, Pierre Dane, Alexa Heekes, Nei-Yuan Hsiao, Mehreen Hunter, Hannah Hussey, Theuns Jacobs, Waasila Jassat, Saadiq Kariem, Reshma Kassanjee, Inneke Laenen, Sue Le Roux, Richard Lessells, Hassan Mahomed, Deborah Maughan, Graeme Meintjes, Marc Mendelson, Ayanda Mnguni, Melvin Moodley, Katy Murie, Jonathan Naude, Ntobeko A.B. Ntusi, Masudah Paleker, Arifa Parker, David Pienaar, Wolfgang Preiser, Hans Prozesky, Peter Raubenheimer, Liezel Rossouw, Neshaad Schrueder, Barry Smith, Mariette Smith, Wesley Solomon, Greg Symons, Jantjie Taljaard, Sean Wasserman, Robert J. Wilkinson, Milani Wolmarans, Nicole Wolter, Andrew Boulle

**Affiliations:** 1 Health Intelligence, Western Cape Government: Health, Cape Town, South Africa; 2 Centre for Infectious Disease Epidemiology and Research, School of Public Health and Family Medicine, University of Cape Town, Cape Town, South Africa; 3 Division of Public Health Medicine, School of Public Health and Family Medicine, University of Cape Town, Cape Town, South Africa; 4 National Department of Health, Pretoria, South Africa; 5 Western Cape Government: Health, Cape Town, South Africa; 6 Groote Schuur Hospital, Western Cape Government: Health, Cape Town, South Africa; 7 Division of Infectious Diseases and HIV Medicine, Department of Medicine, University of Cape Town, Cape Town, South Africa; 8 National Institute for Communicable Diseases, National Health Laboratory Service, Johannesburg, South Africa; 9 School of Public Health, Faculty of Health Sciences, University of the Witwatersrand, Johannesburg, South Africa; 10 Division of Medical Virology, University of Cape Town, Cape Town, South Africa; 11 National Health Laboratory Service, South Africa; 12 Metro Health Services, Western Cape Government: Health, Cape Town, South Africa; 13 Division of Health Systems and Public Health, Department of Global Health, Faculty of Medicine and Health Sciences, Stellenbosch University, Stellenbosch, South Africa; 14 Karl Bremer Hospital, Western Cape Government: Health, Cape Town, South Africa; 15 KwaZulu-Natal Research, Innovation & Sequencing Platform, University of KwaZulu-Natal, Durban, South Africa; 16 Department of Medicine, University of Cape Town, Cape Town, South Africa; 17 Khayelitsha District Hospital, Western Cape Government: Health, Cape Town, South Africa; 18 Mitchells Plain Hospital, Western Cape Government: Health, Cape Town, South Africa; 19 South African Medical Research Council Extramural Unit on Intersection of Noncommunicable Diseases and Infectious Diseases, Cape Town, South Africa; 20 Tygerberg Hospital, Western Cape Government: Health, Cape Town, South Africa; 21 Division of Infectious Diseases, Department of Medicine, Stellenbosch University, Stellenbosch, South Africa; 22 Rural Health Services, Western Cape Government: Health, Cape Town, South Africa; 23 Division of Medical Virology, University of Stellenbosch, Stellenbosch, South Africa; 24 Division of General Medicine, Department of Medicine, Stellenbosch University, Stellenbosch, South Africa; 25 The Francis Crick Institute, London, UK; 26 Department of Infectious Diseases, Imperial College London, London, UK; 27 Wellcome Centre for Infectious Disease Research in Africa, Institute of Infectious Disease and Molecular Medicine, University of Cape Town, Cape Town, South Africa; 28 School of Pathology, Faculty of Health Sciences, University of Witwatersrand, Johannesburg, South Africa

**Keywords:** COVID-19, Omicron, BA.4, BA.5, Death, Severe hospitalization

## Abstract

**Objectives:**

We aimed to compare the clinical severity of Omicron BA.4/BA.5 infection with BA.1 and earlier variant infections among laboratory-confirmed SARS-CoV-2 cases in the Western Cape, South Africa, using timing of infection to infer the lineage/variant causing infection.

**Methods:**

We included public sector patients aged ≥20 years with laboratory-confirmed COVID-19 between May 01-May 21, 2022 (BA.4/BA.5 wave) and equivalent previous wave periods. We compared the risk between waves of (i) death and (ii) severe hospitalization/death (all within 21 days of diagnosis) using Cox regression adjusted for demographics, comorbidities, admission pressure, vaccination, and previous infection.

**Results:**

Among 3793 patients from the BA.4/BA.5 wave and 190,836 patients from previous waves, the risk of severe hospitalization/death was similar in the BA.4/BA.5 and BA.1 waves (adjusted hazard ratio [aHR] 1.12; 95% confidence interval [CI] 0.93; 1.34). Both Omicron waves had a lower risk of severe outcomes than previous waves. Previous infection (aHR 0.29, 95% CI 0.24; 0.36) and vaccination (aHR 0.17; 95% CI 0.07; 0.40 for at least three doses vs no vaccine) were protective.

**Conclusion:**

Disease severity was similar among diagnosed COVID-19 cases in the BA.4/BA.5 and BA.1 periods in the context of growing immunity against SARS-CoV-2 due to previous infection and vaccination, both of which were strongly protective.

## Highlights

• Severe hospitalization or risk of death was similar for BA.4/BA.5 and BA.1 infections.
• Previous infection and vaccination strongly protected against severe COVID-19.
• Growing population immunity against COVID-19 resulted in reduced severe disease.
• Booster vaccinations are important to reduce the public health impact of COVID-19.

## Background

The Omicron SARS-CoV-2 variant of concern has been dominant globally since November 2021, with several sublineages causing surges in infections (Iketani et al*.*, 2022; Tegally et al*.*, 2022; Viana et al*.*, 2022). South Africa experienced an initial large BA.1 infection surge from November 2021 to January 2022. BA.1 was then replaced by BA.2 but with no increase in cases numbers, and this was followed by a BA.4/BA.5 infection surge between April and June 2022 (Tegally et al*.*, 2022; Viana et al*.*, 2022). BA.4 and BA.5 share all mutations with BA.2, except the following: S:69-70del, S:L452R, S:F486V, and S:Q493 (reversion to wild type). In addition, BA.4 is defined by ORF7b:L11F and N:P151S, whereas BA.5 is defined by M:D3N and ORF6:D61 (reversion to wild type) (Das et al*.*, 2022; Dhawan et al*.*, 2022; Kimura et al*.*, 2022; Mohapatra et al*.*, 2022). The combination of mutations in BA.4/BA.5 appear to confer a growth advantage over BA.2, and immune escape from vaccine-derived and BA.1 elicited antibodies (Khan et al*.*, 2022; Tegally et al*.*, 2022). BA.4 and BA.5 infections have been dominant globally since July 2022 (Bedford et al*.*, 2022; Callaway, 2022; UK Health Security Agency, 2022).

We, therefore, compared outcomes of laboratory-confirmed SARS-CoV-2 infections during the April-June 2022 resurgence (proxy for BA.4/ BA.5 infection) with outcomes during each of the four previous waves in South Africa, each of which was caused by a different variant or sublineage, using data on patients with laboratory-confirmed SARS-CoV-2 infection aged ≥20 years using public sector services in the Western Cape Province, South Africa.

## Methods

**Figure 1:**
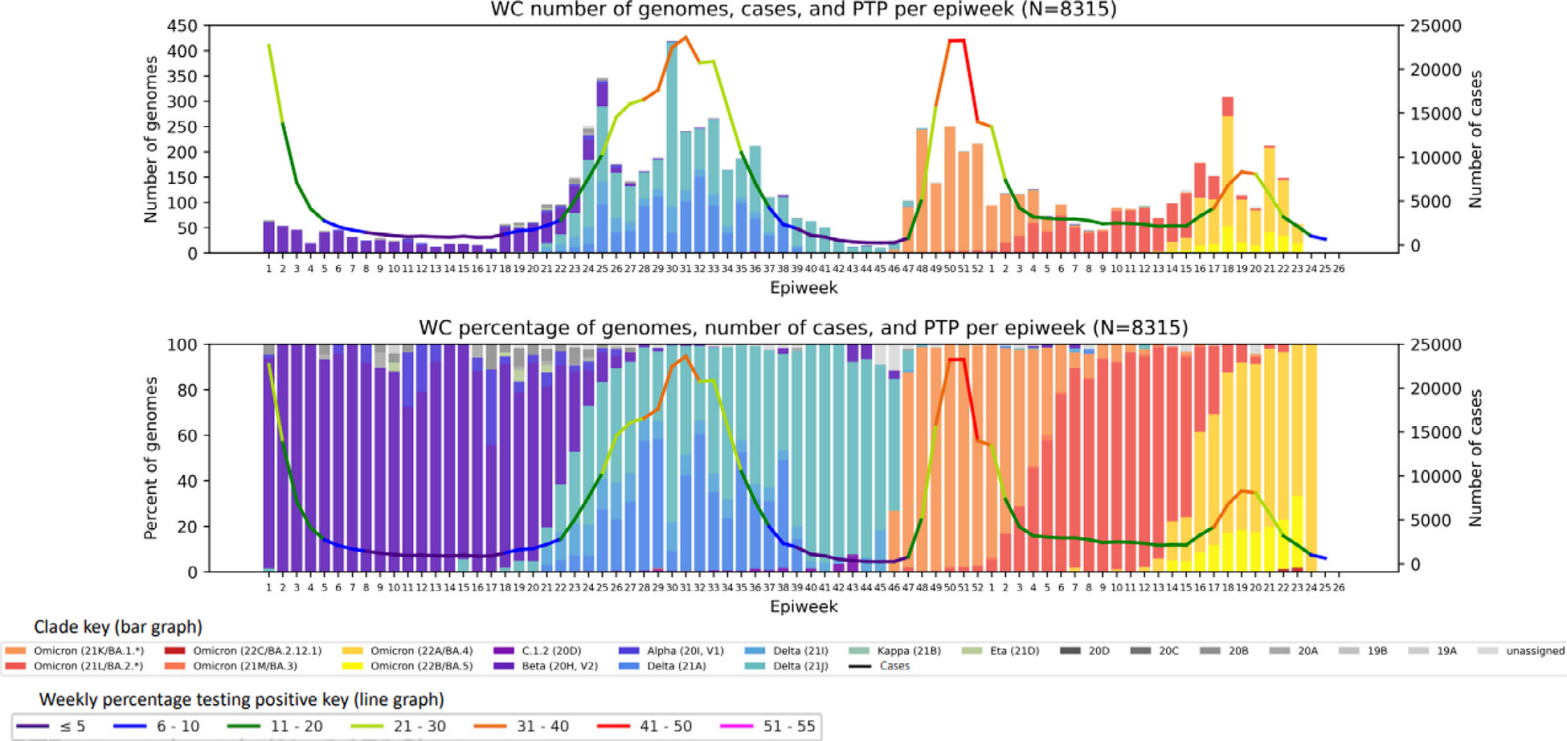
Number of SARS-CoV-2 diagnosed infections, proportion of SARS-CoV-2 tests that are positive (PTP), number of specimens sequenced, and distribution of different SARS-CoV-2 variants and subvariants in the WC, South Africa by epidemiologic week from January 1, 2021, to June 25, 2022. Courtesy Network for Genomics Surveillance in South Africa. PTP, percentage testing positive; WC, Western Cape

We conducted a cohort study using de-identified data from the Western Cape Provincial Health Data Centre (WCPHDC) of public sector patients aged ≥20 years with a laboratory-confirmed COVID-19 diagnosis (positive SARS-CoV-2 polymerase chain reaction (PCR) or antigen test). The Western Cape has nearly 7 million inhabitants, of whom approximately 75% use public sector health services (Western Cape Department of Health, 2020). The WCPHDC and approach for this study have previously been described in detail (Boulle et al*.*, 2019; Davies et al*.*, 2022; Hussey et al*.*, 2022; Western Cape Department of Health in collaboration with the National Institute for Communicable Diseases, 2020). Briefly, for this analysis, waves of infection were defined as starting and ending when the 7-day moving average of public sector COVID-19 hospital admissions exceeded and dropped below 5 and 12 per million population, respectively. We included cases diagnosed from 7 days before the wave start date to 7 days before the wave end date to account for the lag between infection/first symptoms and hospitalization. We thus included data on cases diagnosed from May 1-May 21, 2022, for the BA.4/BA.5 wave, with follow-up through to June 11, 2022. This corresponds to the period when BA.4/BA.5 dominated in the province, accounting for 90% of sequenced cases in the Western Cape (495/548; the remainder were BA.2 [n = 51] with one BA.1 and one recombinant) as shown in Figure 1 (Network for Genomic Surveillance in South Africa, 2022).

We used Cox regression adjusted for age, sex, geographic location, comorbidities, service pressure (number of weekly admissions in the health district) at the time of diagnosis, previously diagnosed infection (≥1 laboratory-confirmed SARS-CoV-2 diagnosis ≥90 days previously), and SARS-CoV-2 vaccination to assess differences in the following COVID-19 outcomes between waves driven by different variants: (i) death and (ii) death or severe hospitalization (admission to intensive care or mechanical ventilation or oral/intravenous steroid prescription). We only included outcomes within 21 days of COVID-19 diagnosis for comparable ascertainment across all waves. All deaths within 21 days of a COVID-19 diagnosis were included unless a clear non-COVID-19 cause of death was recorded. For patients with recorded South African national identity numbers, data are linked to the South African vital registry to identify deaths not recorded in the WCPHDC. Vaccination data were obtained by linking the South African national identifier to the Electronic Vaccine Data System, which records all vaccines administered in the country. The only vaccines available in South Africa to date are BNT162b2 and Ad26.COV2.S. For the regression models, vaccination status was categorized into five groups: (i) “≥3 doses” (three or more homologous or heterologous doses of any vaccine), (ii) “two doses” (two doses of any vaccine), (iii) “single dose Ad26.COV2.S” (single dose of Ad26.COV2.S), (iv) “single dose BNT162b2” (single dose of BNTB162b2), or (v) “unvaccinated”. Participants were considered to be in a particular vaccine group if they had received their last dose ≥7 days before COVID-19 diagnosis for “≥3 doses”, ≥14 days before for “two doses”, and ≥28 days before for the single dose categories.

The study was approved by the University of Cape Town and Stellenbosch University Health Research Ethics Committees and Western Cape Government: Health. Individual informed consent requirement was waived for this secondary analysis of de-identified data.

## Results

We included 3793 patients diagnosed in the BA.4/BA.5 wave and 27,614 (BA.1), 68,715 (Delta), 54,268 (Beta), and 40,204 (ancestral) in waves driven by previous variants (Table 1). The proportion of patients who died within 21 days of COVID-19 diagnosis varied across waves and was 1.9% (BA.4/BA.5), 2.5% (BA.1), 6.4% (Delta), 6.9% (Beta), and 5.3% (ancestral). The proportion with previously diagnosed infection was substantially higher in the BA.4/BA.5 (18.9%) and BA.1 (11.9%) waves compared with previous waves (<3%). In the BA.4/BA.5 wave, 12.9% of COVID-19 cases had received “single dose Ad26.COV2.S” vaccination, 3.9% “single dose BNT162b2”, 36.1% had received “two doses”, and 6.7% had received “≥3 doses”.

**Table 1:** Characteristics and outcomes of COVID-19 cases included from each infection period in the Western Cape.

	Ancestral wave 25 April to 22 July 2020^a^ (n = 40,204)	Beta wave 3 November 2020 to 22 January 2021^a^ (n = 54,268)	Delta wave 30 May to 10 September 2021^a^ (n = 68,750)	BA.1 wave 27 November 2021 to 12 January 2022^a^ (n = 27,614)	BA.4/BA.5 wave 1 May to 21 May 2022^a^ (n = 3793)
**Male sex**	13,380 (33.3%)	19,083 (35.2%)	25,948 (37.7%)	9630 (34.9%)	1327 (35.0%)
					
**Age**					
**20-39 years**	18,720 (46.6%)	21,839 (40.2%)	29,720 (43.2%)	13,944 (50.5%)	1783 (47.0%)
**40-49 years**	8280 (20.6%)	10,594 (19.5%)	14,163 (20.6%)	4905 (17.8%)	767 (20.2%)
**50-59 years**	6982 (17.4%)	10,493 (19.3%)	13,294 (19.3%)	4216 (15.3%)	623 (16.4%)
**60-69 years**	3733 (9.3%)	6929 (12.8%)	6780 (9.9%)	2554 (9.3%)	333 (8.8%)
**≥70 years**	2489 (6.2%)	4413 (8.1%)	4793 (7.0%)	1995 (7.2%)	287 (7.6%)
					
**Noncommunicable diseases**					
**Diabetes**	8265 (20.6%)	11,509 (21.1%)	11,581 (16.9%)	3627 (13.1%)	406 (10.7%)
**Hypertension**	13,065 (32.5%)	19,070 (35.1%)	21,170 (30.8%)	7063 (25.6%)	842 (22.2%)
**Chronic kidney disease**	2013 (5.0%)	2778 (5.2%)	3018 (4.4%)	958 (3.5%)	124 (3.3%)
**Chronic pulmonary disease / asthma**	3099 (7.7%)	4661 (8.6%)	6434 (9.4%)	3040 (11.0%)	411 (10.8%)
					
**Tuberculosis**					
**Previous tuberculosis**	2777 (6.9%)	3450 (6.4%)	4850 (7.1%)	2229 (8.1%)	232 (6.1%)
**Current tuberculosis**	513 (1.3%)	555 (1.0%)	803 (1.2%)	578 (2.1%)	76 (2.0%)
					
**HIV positive**	6203 (15.4%)	5512 (10.2%)	5925 (8.6%)	3298 (11.9%)	307 (8.1%)
					
**Previously diagnosed SARS-CoV-2 infection**	0 (0%)	618 (1.1%)	1798 (2.6%)	3179 (11.5%)	715 (18.9%)
					
**Vaccination**^b^					
**None**	N/A	N/A	63,644 (92.6%)	14,471 (52.4%)	1535 (40.5%)
**Single dose Ad26.COV2.S**	N/A	N/A	2501 (3.6%)	4069 (14.7%)	488 (12.9%)
**Single dose BNT162b2**	N/A	N/A	2289 (3.3%)	1144 (4.1%)	147 (3.9%)
**Two doses Ad26.COV2.S**	N/A	N/A	30 (0.04%)	1127 (4.1%)	298 (7.9%)
**Two doses BNT162b2**	N/A	N/A	286 (0.4%)	6763 (24.5%)	1067 (28.1%)
**Two doses Ad26.COV2.S + BNT162b2**	N/A	N/A	N/A	N/A	5 (0.1%)
**≥3 doses Ad26.COV2.S**	N/A	N/A	N/A	36 (0.1%)	38 (1.0%)
**≥3 doses BNT162b2**	N/A	N/A	N/A	4 (0.01%)	192 (5.1%)
**≥3 doses Ad26.COV2.S + BNT162b2**	N/A	N/A	N/A	N/A	23 (0.6%)
					
**Outcomes within 21 days of diagnosis**					
**Severe admission (not deceased)**^c^	N/A^c^	1916 (3.5%)	2066 (3.0%)	481 (1.7%)	61 (1.6%)
**Death**	2147 (5.3%)	3717 (6.9%)	4368 (6.4%)	699 (2.5%)	70 (1.9%)

N/A, not applicable.

a Date of diagnoses for cases included in each wave. We included cases diagnosed from 7 days before the "wave start" to the date of "wave end" (deemed to occur when 7-day moving average of daily new public sector admissions exceeded 5 million [start] and dropped below 12 million [end] respectively).

b Vaccination is summarized as vaccine type and number of doses provided diagnosis was ≥28 days after first dose, ≥14 days after second dose, and ≥7 days after third dose;

c Admission to an intensive care unit, mechanical ventilation, or prescription of oral or intravenous steroids; not reported for wave one as steroids not widely used until after June 16, 2020.

The adjusted hazard of severe hospitalization or death in the BA.4/BA.5 wave was similar to the BA.1 wave (adjusted hazard ratio [aHR] 1.12; 95% confidence interval [CI]: 0.93; 1.34) (Table 2). Both Omicron-driven waves had lower hazards of severe hospitalization or death than previous waves (Table 2). Previously diagnosed infection was strongly protective against severe hospitalization or death (aHR 0.29; 95% CI 0.24; 0.36), as was vaccination with aHR (95% CI) of 0.17 (0.07; 0.40); 0.37 (0.33; 0.42); 0.26 (0.21; 0.32) and 0.61 (0.56; 0.67) for “≥3 doses”, “two doses”, “single dose Ad26.COV2.S”, and “single dose BNT162b2”, respectively. In a model not adjusting for vaccination and previously diagnosed infection, the hazard of severe hospitalization or death in the BA.4/BA.5 vs BA.1 waves was reduced compared with the fully adjusted model (aHR 0.90; 95% CI: 0.75; 1.08). In an analysis restricted to the BA.4/BA.5 period, previously diagnosed infection remained strongly protective against severe hospitalization or death (aHR 0.23; 95% CI 0.10; 0.52) as did vaccination, except for “single dose BNT162b2” (aHR [95% CI]: 0.20 [0.08; 0.49], 0.39 [0.25; 0.59], 0.51 [0.27; 0.99] and 0.94 [0.44; 1.99] for “≥3 doses”, “two doses”, “single dose Ad26.COV2.S”, and “single dose BNT162b2”, respectively. Results were all similar when examining the outcome of death alone.

**Table 2:** Associations between different infection periods and severe COVID-19 outcomes adjusted for patient characteristics, sub-district, vaccination, and previously diagnosed infection using Cox regression.

	Outcome = death; not adjusted for vaccination and previous infection		Outcome = death; adjusted for vaccination and previous infection		Outcome = severe hospitalization^a^/death; not adjusted for vaccination or previously diagnosed infection		Outcome = severe hospitalization^a^/death; adjusted for vaccination and previously diagnosed infection	
	Adjusted^b^ HR	95% CI	Adjusted HR	95% CI	Adjusted^b^ HR	95% CI	Adjusted HR	95% CI
**Male sex (vs female)**	1.40	1.34; 1.45	1.40	1.34; 1.45	1.27	1.23; 1.31	1.26	1.22; 1.30
**Age (vs 20-39 years)**								
**40-49 years**	2.54	2.30; 2.81	2.57	2.33; 2.84	2.00	1.87; 2.15	2.04	1.90; 2.19
**50-59 years**	5.46	4.99; 5.97	5.56	5.08; 6.08	3.42	3.21; 3.65	3.50	3.28; 3.74
**60-69 years**	12.55	11.47; 13.73	12.88	11.77; 14.10	6.39	5.97; 6.83	6.56	6.13; 7.01
**≥70 years**	23.19	21.15; 25.43	23.93	21.82; 26.24	10.35	9.65; 11.09	10.65	9.94; 11.42
**Comorbidities (vs comorbidity absent)**								
**Diabetes**	2.01	1.92; 2.10	2.01	1.93; 2.10	1.97	1.89; 2.04	1.98	1.91; 2.06
**Hypertension**	1.08	1.03; 1.13	1.07	1.02; 1.12	1.18	1.14; 1.23	1.17	1.13; 1.22
**Chronic kidney disease**	1.90	1.80; 2.00	1.90	1.81; 2.00	1.63	1.56; 1.70	1.63	1.56; 1.70
**Chronic pulmonary disease / asthma**	0.98	0.93; 1.04	0.99	0.93; 1.04	1.18	1.13; 1.23	1.19	1.14; 1.24
**Previous tuberculosis**	1.30	1.20; 1.40	1.28	1.19; 1.38	1.25	1.17; 1.33	1.23	1.16; 1.31
**Current tuberculosis**	2.53	2.20; 2.91	2.44	2.13; 2.81	2.89	2.59; 3.23	2.79	2.50; 3.11
**HIV**	1.60	1.48; 1.72	1.60	1.49; 1.72	1.54	1.45; 1.64	1.54	1.45; 1.64
**Number of admissions in district in week of diagnosis (vs <1/3 of maximum)**								
**1/3 to <2/3**	1.11	1.05; 1.17	1.12	1.06; 1.18	1.03	0.98; 1.08	1.04	0.99; 1.09
**≥2/3**	1.12	1.05; 1.20	1.13	1.06; 1.21	1.05	0.99; 1.11	1.06	1.00; 1.12
**Previously diagnosed SARS-CoV-2 infection**								
**Yes (vs none)**			0.51	0.42; 0.63			0.29	0.24; 0.36
**Vaccination (vs None)**^c^								
**Single dose BNT162b2**			0.56	0.49; 0.63			0.61	0.56; 0.67
**Single dose Ad26.COV2.S**			0.24	0.18; 0.33			0.26	0.21; 0.32
**Two doses (Ad26.COV2.S and/or BNT162b2)**			0.36	0.31; 0.42			0.37	0.33; 0.42
≥ 3 doses **(≥ 3 doses Ad26.COV2.S and/or BNT162b2)**			0.06	0.01; 0.40			0.17	0.07; 0.40
**Wave period (dominant variant)**								
**Wave 1 (ancestral)**	2.08	1.90; 2.28	1.30	1.17; 1.44	N/A^a^		N/A^a^	
**Wave 2 (Beta)**	2.35	2.16; 2.57	1.47	1.34; 1.62	2.06	1.93; 2.20	1.28	1.20; 1.38
**Wave 3 (Delta)**	2.58	2.37; 2.81	1.75	1.59; 1.92	2.16	2.03; 2.29	1.44	1.35; 1.54
**Wave 4 (Omicron BA.1)**	Ref		Ref		Ref		Ref	
**Wave 5 (Omicron BA.4/BA.5)**	0.93	0.72; 1.20	1.16	0.90; 1.50	0.90	0.75; 1.08	1.12	0.93; 1.34

CI, confidence interval; HR, hazard ratio; N/A, not applicable; Ref, reference.

a Admission to an intensive care unit, mechanical ventilation, or prescription of oral or intravenous steroids; not reported for wave one as steroids not widely used until after June 16, 2020.

b Adjusted for all variables shown in the table and sub-district/district, but not for vaccination or previously diagnosed infection.

c Vaccination status is categorized as “single dose BNT162b2” (≥28 days after single dose BNT162b2), “single dose Ad26.COV2.S” (≥28 days after single dose Ad26.COV2.S), “two doses” (≥14 days after second dose of homologous or heterologous vaccination with Ad26.COV2.S and/or BNT162b2), and “≥3 doses” (≥7 days after third dose of homologous or heterologous vaccination with Ad26.COV2.S and/or BNT162b2).

## Discussion

Using the period of diagnosis as a proxy for being infected with different Omicron sublineages in the Western Cape, we found no difference in the risk of severe COVID-19 hospitalization or death during the BA.4/BA.5 period compared to the BA.1 period, both of which had better outcomes than previous waves. Strong protection against severe COVID-19 conferred by previous infection and vaccination was retained in the BA.4/BA.5 wave, with three homologous doses of Ad26.COV2.S or BNT162b2 or a heterologous combination of these provides 83% protection (95% CI 60; 93%) against severe COVID-19 hospitalization or death among laboratory-confirmed cases.

A study in animals recently suggested that BA.4/BA.5 may be more pathogenic than BA.2 (Kimura et al*.*, 2022). Although we did not compare BA.4/BA.5 with BA.2 directly, as BA.2 did not cause a distinguishable surge in infections in the Western Cape, disease severity of BA.2 and BA.1 are similar (Lewnard et al*.*, 2022), and we found no evidence of worse clinical outcomes with BA.4/BA.5 compared to BA.1. Nonetheless, our findings need to be interpreted in the context of South African SARS-CoV-2 epidemiology with progressively increasing seroprevalence due to previous infection (mostly undiagnosed) and/or vaccination (Bingham et al*.*, 2022; Madhi et al*.*, 2022; Sun et al*.*, 2022). For example, among blood donors, after the BA.1 wave, the estimated national prevalence of anti-nucleocapsid antibodies was 87% (indicating previous infection), with a further 10% having anti-spike antibodies only (suggesting vaccination without previous infection) (Bingham et al*.*, 2022). Since anti-nucleocapsid antibodies have lower sensitivity for identifying previous infections and may wane, it is possible that previous exposure to SARS-CoV-2 infections and/or vaccination may even exceed 97%. Indeed, our finding that the aHR shifted toward a lower risk of severe outcomes during BA.4/BA.5 vs BA.1 in models not accounting for vaccination and previously diagnosed infection suggests that the observed continued ecologic decoupling of COVID-19 cases and severe outcomes, is at least partly due to growing protection against severe disease from both previous infection and vaccination. The observed clinical outcomes of infection with BA.4/BA.5 may therefore be different in settings with different previous variant infection and vaccination exposure. With the progression of the SARS-CoV-2 pandemic globally, it is increasingly difficult to determine the clinical severity of any variant in a completely naïve individual. However, for health service planning, this is less relevant than the real-world effect in populations with varying degrees of immune protection (Mefsin et al*.*, 2022). For example, although we showed a similar risk of severe hospitalization or death in the BA.4/BA.5 and BA.1 waves when adjusted for vaccination and previously diagnosed infection, the actual burden of admissions and deaths was much lower in the BA.4/BA.5 waves, with the peak 7-day moving average of admissions and deaths being 222 and 36 in the BA.1 wave vs 66 and nine in the BA.4/BA.5 wave. The ability to use routine data to rapidly assess the relative severity of waves caused by different lineages and variants adjusted for comorbidities, vaccination and previous infection has been especially valuable for local health service planning (Davies et al*.*, 2022).

To our knowledge, this is one of the first comparisons of the clinical severity of BA.4/BA.5 infections with previous variants with relatively complete adjustment for comorbidities and vaccination among all diagnosed cases. Nonetheless, this type of data and analysis has several limitations, which have been described in detail previously (Davies et al*.*, 2022). These include using the time of infection as a proxy for the variant causing infection rather than actual genomic sequencing or PCR test proxies (Wolter et al*.*, 2022) which would be more accurate, could allow assessing the biological effect associated with specific mutations and would overcome challenges with comparing disease severity across waves due to differences in testing practices, treatment availability, and health service pressures. Notably, testing in the BA.4/BA.5 wave was at the lowest levels since the start of the pandemic with less testing of patients with milder disease; hence we may have over-estimated disease severity in this wave. For example, the peak weekly testing rate in the BA.4/BA.5 wave in the Western Cape was only 1/3 of that during the BA.1 wave (256 vs 756 tests per week per 100,000 population). Although we would have liked to assess the effects of time since vaccination and homologous vs heterologous vaccine doses, it was not possible to do this analysis due to small numbers of participants with each of the different vaccine combinations and durations since the last dose (Lyke et al*.*, 2022). The routine healthcare data used did not allow us to distinguish between severe hospitalizations and deaths where the diagnosis of COVID-19 may have been incidental or contributory rather than causal. We also had incomplete ascertainment of key covariates, especially previously diagnosed infection, due to substantial missed diagnoses (only 19% of our BA.4/BA.5 cases had previously diagnosed infection, whereas seroprevalence studies suggest at least 87% of the population had previous infection before the BA.4/BA.5 wave) (Bingham et al*.*, 2022) and only including infections that were diagnosed more than 90 days apart. Similarly, due to the small numbers of patients with previously diagnosed infection and severe disease in the BA.4/BA.5 wave (n = 6), we were unable to assess whether there were differences in the extent of protection conferred by previous infection with different variants. Even in those with previously diagnosed infection it is possible that they had additional unascertained infections in other waves that may have impacted on their protection against severe disease due to BA.4/BA.5. Further, we had no data on vaccinations received outside of the province or without submitting a South African identity number and no data on undiagnosed comorbidities as we can only adjust for those algorithmically identified in the WCPHDC.

In conclusion, we found similar disease severity among diagnosed COVID-19 cases in the BA.4/BA.5 and BA.1 periods, both of which were associated with less severe outcomes than waves caused by previous SARS-CoV-2 variants. This finding is in the context of growing immunity against SARS-CoV-2 with strong protection against severe outcomes conferred by previous infection and vaccination, especially >3 doses. Three homologous doses of Ad26.COV2.S or BNT162b2 or a heterologous combination provided 83% protection (95% CI 60; 93%) against severe COVID-19 hospitalization or death among laboratory-confirmed cases. Ensuring that individuals at high risk of severe COVID-19 outcomes have at least three vaccine doses remains a key strategy to limit the public health impact of further COVID-19 waves. Further research is needed to understand the specific differences in viral phenotype caused by the mutations in BA.4 and BA.5, as these mutations may occur in future variants and subvariants. In addition, it would be useful to quantify the protection provided by different types of immunity, such as natural infection with different variants, hybrid immunity (natural infection with vaccination), and heterologous versus homologous vaccination and waning of immunity.

## Declaration of competing interest

All authors have no competing interests to declare.

## Funding

We acknowledge funding for the Western Cape Provincial Health Data Centre (WCPHDC) from the Western Cape Department of Health, the US National Institutes for Health (R01 HD080465, U01 AI069924), the Bill and Melinda Gates Foundation (1164272, 1191327), the United States Agency for International Development (72067418CA00023), the European Union (101045989) and the Grand Challenges International COVID-19 Data Alliance (ICODA) pilot initiative delivered by Health Data Research UK and funded by the Bill & Melinda Gates and Minderoo Foundations (INV-017293). Funding was also received from Wellcome (203135/Z/16/Z [RJW, GM, WCPHDC], 222574 [RJW, WCPHDC] 214321/Z/18/Z [GM]) and the Medical Research Council of South Africa (RJW, MAD). RJW additionally receives support from the Francis Crick Institute, which is funded by Wellcome (FC0010218), MRC (UK) (FC0010218), and Cancer Research UK (FC0010218). GM is also funded by the South African Research Chairs Initiative of the Department of Science and Technology and National Research Foundation (NRF) of South Africa (Grant No 64787). The funders had no role in the study design, data collection, data analysis, data interpretation, or writing of this report. The opinions, findings, and conclusions expressed in this manuscript reflect those of the authors alone. For the purposes of open access, the author has applied CC-BY public copyright to any author-accepted version arising from this submission.

## Ethical approval

The study was approved by the University of Cape Town and Stellenbosch University Health Research Ethics Committees and Western Cape Government: Health. Individual informed consent requirement was waived for this secondary analysis of de-identified data.
